# Analyzing the Automatic Power Level Control Effect of a Signal Generator in RF Power Sensor Calibration by a Direct Comparison Transfer Method and a Millimeter Wave Application

**DOI:** 10.3390/s24020609

**Published:** 2024-01-18

**Authors:** Erkan Danaci

**Affiliations:** TUBITAK National Metrology Institute (UME), P.O. Box 54, Kocaeli 41470, Türkiye; erkan.danaci@tubitak.gov.tr; Tel.: +90-262-679-50-00

**Keywords:** automatic level control of RF source, direct comparison transfer method, coaxial RF power sensor calibration, millimeter wave power sensor calibration

## Abstract

Most calibration laboratories prefer the Direct Comparison Transfer Method (DCTM) for a reliable and accurate calibration of power sensors in the radio frequency (RF) scope. Most studies suggest using this calibration method, with its automatic power level control (APLC) of RF signal generators. The APLC is preferred to keep the output power level of the signal generator the same, while the power sensor is calibrated and the reference power sensor is connected to the measurement system. The known APLC mechanisms are also explained for the DCTM, and a comparison of the calibration factor values carried out with and without the automatic power level control process in the DCTM is also given in this study. RF power sensor calibrations with coaxial and waveguide connector types are examined with DCTM in this study as well. This study shows that the DCTM, unless with APLC, should be applied for the waveguide power sensor’s calibration at millimeter wave frequencies.

## 1. Introduction

Radio Frequency (RF) power sensors are indispensable components for calibration laboratories for accurate RF power measurements. RF power sensors never display actual power values accurately because of their many losses. These losses are categorized as impedance mismatch, reflection, insertion, dielectric, and RF to direct current conversion losses [1,2]. Insertion, dielectric, and RF-directed current conversion losses are often referred to as losses in power sensors [3]. These losses stem from the sensor design and are inherent to the RF power sensors.

In order to accurately measure the RF power applied to the RF power sensors, different power sensor calibration procedures have been developed at primary and secondary levels. A primary-level micro-calorimeter system characterizes the power sensors. For the secondary level calibration, comparative measurements are employed using power sensors with well-known characteristics [4,5,6].

There are two parameters obtained from the calibration methods for the characterization of power sensors. These parameters are called Effective Efficiency (EE) and Calibration Factor (CF). The EE parameter is known as the ratio of the power sensor’s output power to the consumed power in the power sensor. The EE parameter is obtained in the calibration performed with the primary level micro-calorimeter system [7,8,9]. The CF parameter is known as the ratio of the power sensor’s output power to the input power of the power sensor. The comparative calibration methods obtain the calibration factor of power sensor [7,8,9,10]. The relation between the CF and the EE is given in Equation (1).

(1) $$\mathrm{CF}=\left(1-\rho^{2}\right)\cdot \eta$$

where ρ is the magnitude of the reflection coefficient of the power sensor’s connector, and η is the EE of the power sensor.

There are many power sensor types, such as thermistor mount, thermocouple, and diode. Thermal sensors such as thermistor mounts that measure RF power with thermal changes against RF signals are characterized in the micro-calorimeter systems. Since most of the RF and/or microwave devices used as power sensors at frequencies above 110 GHz operate based on thermal principles, new approaches are being developed for the primary level characterization of RF power sensors above 110 GHz frequencies [11].

**Figure 1:**
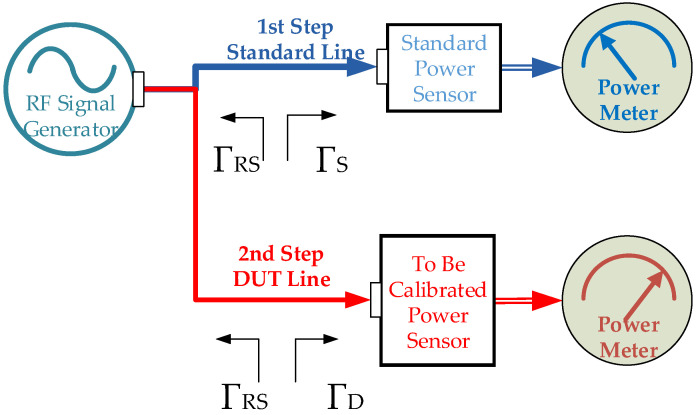
Calibration setup for SM of RF power sensor.

**Figure 2:**
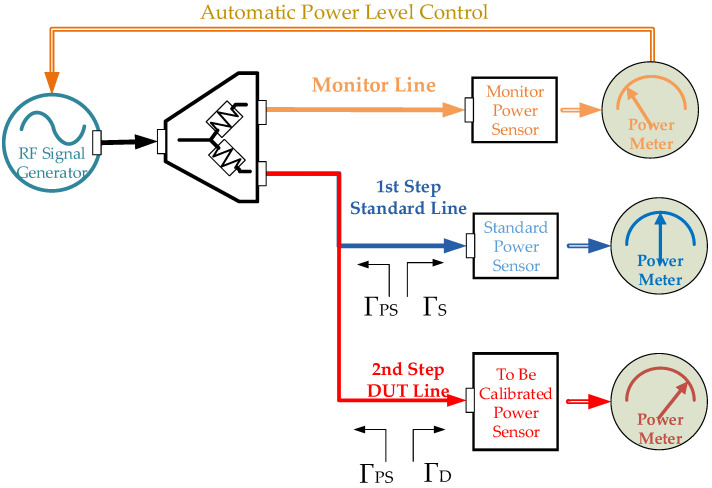
Calibration setup for DCTM of RF power sensor.

Both thermal principle-based and diode-type power sensors can be characterized by using comparative measurements in RF power sensor calibrations [12,13]. The calibration laboratories widely prefer two methods in power sensor characterizations as comparative methods. These methods are known as the Direct Comparison Transfer Method (DCTM) and the Substitution Method (SM) [14,15,16]. Figure 1 and Figure 2 give the basic setups for SM and DCTM, respectively.

There are fewer components in the SM setup compared to the DCTM setup. The output power from the signal generator is assumed to be the same at the input of the DUT and the STD power sensor connection in the SM setup. The power value shift is ignored in the SM setup, and the effect of impedance mismatch in determining the CF is ignored if a well-characterized attenuator/adapter at the output of the RF signal source is not used in the SM setup. The DCTM needs an experienced operator. The operator should control the output power level of the RF signal generator during STD and DUT power measurements in the DCTM.

The advantages and disadvantages of the two power sensor calibration methods based on comparative measurements are summarized in Table 1 [17].

In this study, the known Automatic Power Level Control (APLC) mechanisms are summarized for the DCTM, and the DCTM without APLC application is suggested. In order to show the DCTM application without the APLC method, the calibration of RF power sensors of different connector types, such as coaxial and waveguide (WG) with the DCTM, are investigated in this study. In addition, the comparison of the CF values as a result of the power sensor calibration with the DCTM measurement method with and without APLC is given with different power levels.

## 2. The DCTM Measurement Setups and Circuit Components for Different Types Power Sensors

### 2.1. The DCTM Measurement Setups for Coaxial Power Sensors

In the DCTM, a power splitter is placed between the RF signal generator and the power sensors. Using a power splitter, the operator can continuously monitor the output port of the RF signal generator with another power sensor (MON). Thus, the power drift effect of the signal source can be eliminated when the standard power sensor (STD), with a known CF value, and the device under a test power sensor (DUT), which is an unknown CF value, are connected to the other output port of the power splitter, respectively. The operator simultaneously reads the powers from both power meters (MON and STD/DUT) behind the power sensors. In this method, it is possible to calculate the CF value of power sensors by measuring them in two steps. In the first step, the MON and STD powers are read, and in the second step, the MON and DUT powers are read. For this type of multi-instrument measurement in a broad frequency spectrum, the calibration laboratories should use specially developed software, and a computer should be used for the DCTM.

The impedance mismatch is calculated using the equivalent impedance of the output ports of the power splitter and power sensors’ reflection coefficients in the DCTM. The accurate impedance mismatch calculation is impossible when one of the RF connectors’ reflection coefficient is unknown in the vector. The majority of manufacturers of RF signal sources only provide the output port’s magnitude reflection coefficient or the standing wave ratio of the output connectors. In the SM, the operator connects the power sensors directly to the RF signal source output connector. So, it is impossible to calculate the accurate impedance mismatch in the SM. The DCTM was developed for the SM’s impedance mismatch calculation problem and the RF signal generator’s output power level drifting problem. Calibration laboratories can calculate the impedance mismatch errors by using the DCTM.

Equation (2) gives the CF of the DUT (CF_DUT_) calculation for DCTM.

(2) $$CF_{DUT}=CF_{STD}\cdot \frac{P_{D}}{P_{S}}\cdot \frac{P_{mS}}{P_{mD}}\cdot \frac{\left(1+{\left|\Gamma_{D}\right|}^{2}\cdot {\left|\Gamma_{PS}\right|}^{2}-2\cdot \left|\Gamma_{D}\right|\cdot \left|\Gamma_{PS}\right|\cdot \mathrm{Cos}\left(\Theta_{D}+\Theta_{PS}\right)\right)}{\left(1+{\left|\Gamma_{S}\right|}^{2}\cdot {\left|\Gamma_{PS}\right|}^{2}-2\cdot \left|\Gamma_{S}\right|\cdot \left|\Gamma_{PS}\right|\cdot \mathrm{Cos}\left(\Theta_{S}+\Theta_{PS}\right)\right)}$$

where CF_DUT_ is unknown CF values of the DUT power sensor, CF_STD_ is known CF values of the STD power sensor, P_D_ is the measured power at the DUT line, P_S_ is the measured power at the standard line, P_mD_ is the measured power at the monitor line when the DUT is connected to the measurement setup, P_mS_ is the measured power at the monitor line when the STD is connected to the measurement setup, Γ_D_ is the complex reflection coefficient of the DUT power sensor’s RF connector, Γ_S_ is the complex reflection coefficient of the STD power sensor’s RF connector, Γ_PS_ is the conjugate reflection coefficient of the STD and DUT connecting port of the power splitter, Θ_D_ is the phase of Γ_D_, Θ_S_ is the phase of Γ_S_, and Θ_PS_ is the phase of Γ_PS_.

### 2.2. The DCTM’s Measurement Setups for Waveguide Power Sensors

**Figure 3:**
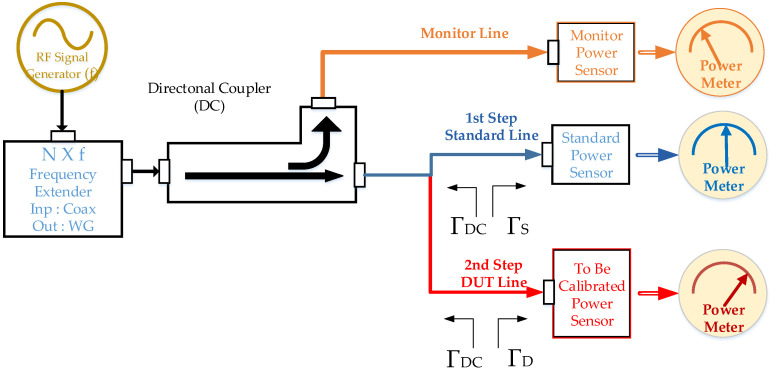
Functional diagram of WG power sensor calibration setup for DCTM.

Given the pros and cons of both comparative power sensor calibration methods, it is suggested that the DCTM method for coaxial and waveguide (WG) power sensor calibrations might be preferred over the SM. Figure 3 shows the DCTM method for the WG power sensor.

In the calibrations to be carried out for the CF values of both coaxial and WG power sensors, the operator needs an RF signal generator and a circuit element splitting the RF signal source’s output into two branches. The power splitter is preferred for the coaxial power sensor calibration setup. The directional coupler (DC) is preferred for the WG power sensor calibration setup. The MON power sensor is used for both connector-type setups to observe how much the output power of the RF power changes when the DUT power sensor or the STD power sensor is connected to the measurement setup in the DCTM setup.

There is a frequency extender in Figure 3. A frequency extender is an optional device that is used to access the millimeter wave frequencies. The proper power meters are used in WG power sensor calibration setups. The CF of the DUT power sensor can be calculated similarly using Equation (2). It is necessary to replace Γ_PS_ and Θ_PS_ with Γ_DC_ and Θ_DC_ in Equation (2) for the CF_DUT_ calculation in the WG DCTM.

### 2.3. The Automatic Power Level Controls at the DCTM’s Setups

Most publications suggest using the APLC connection for power level stability at the application, as shown in Figure 2 [18,19,20]. The DCTM prefers the APLC connection for keeping the output power at the same level, either when the DUT or the STD are connected to the measurement system.

The operator can perform two types of APLC, given below, in the DCTM.

I. APLC can be performed by applying the MON power meter output to the automatic power control input of the RF signal source as the desired power value (as given in Figure 2). The RF signal source must have the APLC option in the first automatic power control method. In this method, the instantaneous power level is adjusted at the time of measurement, depending on the response time of the RF signal source at the time of measurement.
II. APLC can be performed by scanning all frequencies for the desired power value at the power splitter’s output; preliminary measurements are performed on the MON line, ensuring that the RF signal source delivers the same power level at all frequencies. If the RF signal source does not have the APLC option, the operator must use this APLC method. In this case, preliminary measurements are performed before starting the actual DUT calibration measurements to determine the power level for each frequency of the RF signal source.

Both APLC methods are used to protect the STD and DUT power sensors from applying the wrong power levels during calibration.

Frequency extenders are used to generate RF signals for frequencies above 110 GHz at millimeter-wave frequencies and WG systems [21,22]. The operator can apply a fixed power level to the input of the frequency multiplexers from coaxial output signal sources. The operator can obtain only a fixed output power at the output of extenders as written in the specifications given by the manufacturers. Most extenders do not have a suitable feedback input to perform the APLC. Therefore, the operator can apply only the second APLC method described above in calibrating the WG power sensors at frequencies above 110 GHz in the DCTM.

## 3. APLC Impact Analysis in the DCTM

In this study, measurements with APLC and without APLC were carried out with the DCTM’s measurement setup established for coaxial power sensors. The experimental measurements were repeated for two different power sensors at two different power levels. To show how the DCTM is applicable without APLC at the WG power sensor, a WG power sensor calibration was performed, and the calibration results are shared in this study too.

### 3.1. Coaxial Power Sensor Calibration by the DCTM at 0 dBm

In the first experimental measurement, 0 dBm (1 mW) was preferred as the power level, and the devices which were owned of TÜBİTAK UME were used. The Boonton 51011 model coaxial power sensor was used as the DUT power sensor with the proper power meter. An E8257D (Keysight) model RF signal generator was used as the source, N8481A (Keysight) as the reference power sensor (STD) with a N1914A (Keysight) power meter, N8481A (Keysight) as the monitor (MON) power sensor with a N1914A (Keysight) power meter, and 1870A (Weinschel) as the power splitter were used for determining the CF of the DUT power sensor. The measurement was performed according to Figure 2’s setup with a special developed software (CF Software, R1.0, TÜBİTAK UME), and uncertainty calculations were carried out [17].

To perform the measurement with APLC, power values were determined by preliminary measurements to ensure that the output power of the RF signal generator is the same at determined frequencies. Before the measurement, some parameters were chosen, such as 10 repetitive measurements with 1 (one)-second intervals at each frequency and a waiting time for power level stabilization between frequencies of 120 s. To determine the effect of the RF connector error on the measurement, the operator rotated the connectors of the DUT and STD power sensor three times at 120° intervals, and performed three measurements. Measurement results with APLC are given in Table 2.

The measurement results of the Boonton 51011 DUT power sensor calibration using the DCTM performed at the same condition, with the same reference power sensor, and with the same parameters without APLC are given in Table 2.

The calculated CF uncertainties of the DUT power sensor with the Law of Propagation method, according to the International Guide to the Expression of Uncertainty in Measurement (GUM) document [23], for the DCTM with APLC and the DCTM without APLC are also given in Table 2.

**Figure 4:**
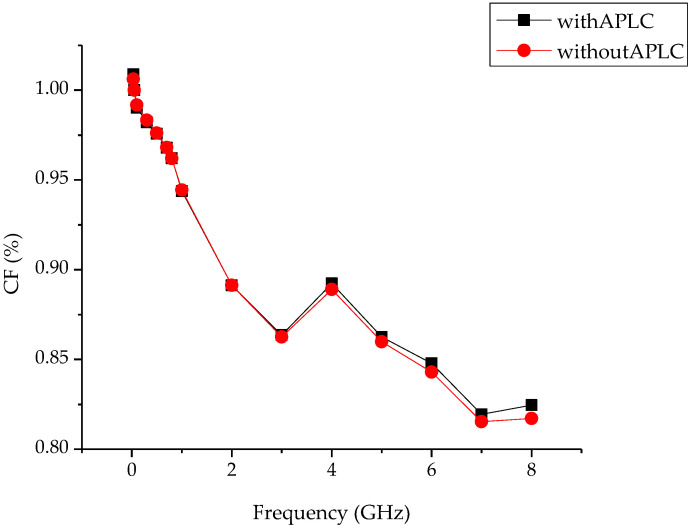
CF values of Boonton 51011 with and without APLC for 0 dBm.

The CF values of the DCTM with APLC and that without the APLC of DUT are given in Figure 4 for the 0 dBm reference power level.

Uncertainties were calculated according to the model function given in Equation (3), and are given in Table 2 with a coverage factor of two (95%).

(3) $$CF_{DUT}=CF_{STD}\cdot \frac{\left(P_{D}+\delta PM_{D}\right)}{\left(P_{S}+\delta PM_{S}\right)}\cdot \frac{\left(P_{mS}+\delta PM_{mS}\right)}{\left(P_{mD}+\delta PM_{mD}\right)}\cdot \frac{\left(1+{\left|\Gamma_{D}\right|}^{2}\cdot {\left|\Gamma_{PS}\right|}^{2}-2\cdot \left|\Gamma_{D}\right|\cdot \left|\Gamma_{PS}\right|\cdot \mathrm{Cos}\left(\Theta_{D}+\Theta_{PS}\right)\right)}{\left(1+{\left|\Gamma_{S}\right|}^{2}\cdot {\left|\Gamma_{PS}\right|}^{2}-2\cdot \left|\Gamma_{S}\right|\cdot \left|\Gamma_{PS}\right|\cdot \mathrm{Cos}\left(\Theta_{S}+\Theta_{PS}\right)\right)}$$

where δPM_D_ is the uncertainty of the DUT power meter, δPM_S_ is the uncertainty of the STD power meter, δPM_mD_ is the uncertainty of the MON power meter at the DUT that was connected to the setup, δPM_mS_ is the uncertainty of the MON power meter at the STD that was connected to the setup.

From the uncertainty calculations given in Table 2, the effect distributions on the combined uncertainty values of the parameters with APLC uncertainty and without APLC uncertainty calculations for 50 MHz frequency are shown in Table 3 and Table 4, respectively.

Among the uncertainty components used to calculate the uncertainty value of CF of the DUT power sensor given in Table 3, it is seen that the biggest contribution to the combined uncertainty comes from the CF of the STD power sensor. The components coming from the read power values affect the combined uncertainty less, while the uncertainties coming from the reflection coefficient magnitude and phases are the components that affect the combined uncertainty the least.

Among the uncertainty components used to calculate the uncertainty value of CF of the DUT power sensor given in Table 4, it is seen that the biggest contribution to the combined uncertainty comes from the CF of the STD power sensor, as is similar in Table 3.

### 3.2. The Coaxial Power Sensor Calibration by the DCTM at −30 dBm

In the second experimental, the devices which were owned of TÜBİTAK UME were used. The RF power was fixed to −30 dBm, and the HP 8484A model coaxial power sensor was used as a DUT power sensor with the proper power meter. The E8257D (Keysight) model RF signal generator as the source, NRPT18T (R and S) as the reference power sensor (STD) with the N1914A (Keysight) power meter, the N8481A (Keysight) as the monitor power sensor (MON) with the N1914A (Keysight) power meter, and the 1870A (Weinschel) as the power splitter were used for determining the CF of the DUT power sensor. The measurement setup was performed according to Figure 2’s setup, and uncertainty calculations were carried out.

To perform the measurement with APLC, power values were determined by preliminary measurements to ensure that the output power of the RF signal generator was the same at the determined frequencies with a special developed software (CF Software, R1.0, TÜBİTAK UME). Ten repetitive measurements with 1 (one)-second intervals at each frequency were performed. The waiting time for power level stabilization between frequencies was 120 s. In order to determine the effect of the RF connector error on the measurement, the operator rotated the connectors of the DUT and the STD power sensor three times at 120° intervals, and performed three measurements. The results with APLC are given in Table 5.

The measurement results of the HP8484A DUT power sensor calibration by the DCTM, which was performed with the same condition, with the same reference power sensor, and with the same parameters without APLC, are given in Table 5.

The DUT CF uncertainties calculated with the Law of Propagation method according to GUM for the DCTM without APLC and with APLC are also in Table 5 for the −30 dBm power level.

Uncertainties were calculated according to the model function given in Equation (4) and are given in Table 5 with coverage a factor of two (95%).

(4) $$CF_{\mathrm{DUT}}=CF_{\mathrm{STD}}\cdot \frac{\left(P_{D}+\delta PM_{D}\right)}{\left(P_{S}+\delta PM_{S}\right)}\cdot \frac{\left(P_{\mathrm{mS}}+\delta PM_{\mathrm{mS}}\right)}{\left(P_{\mathrm{mD}}+\delta PM_{\mathrm{mD}}\right)}\cdot \frac{1}{{\left|S_{21}\right|}^{2}}\cdot \frac{\left(1+{\left|\Gamma_{D}\right|}^{2}\cdot {\left|\Gamma_{\mathrm{PS}}\right|}^{2}-2\cdot \left|\Gamma_{D}\right|\cdot \left|\Gamma_{\mathrm{PS}}\right|\cdot \mathrm{Cos}\left(\Theta_{D}+\Theta_{\mathrm{PS}}\right)\right)}{\left(1+{\left|\Gamma_{S}\right|}^{2}\cdot {\left|\Gamma_{\mathrm{PS}}\right|}^{2}-2\cdot \left|\Gamma_{S}\right|\cdot \left|\Gamma_{\mathrm{PS}}\right|\cdot \mathrm{Cos}\left(\Theta_{S}+\Theta_{\mathrm{PS}}\right)\right)}$$

where S_21_ is the forward transmission coefficient of the attenuator used before the DUT power sensor.

From the uncertainty calculations given in Table 5, the effect of the distributions on the combined uncertainty value of the parameters with APLC uncertainty and without APLC uncertainty calculations for 50 MHz frequency are shown in Table 6 and Table 7, respectively.

Among the uncertainty components in Table 6, it is seen that the biggest contribution to the combined uncertainty comes from the CF of the STD power sensor. The components coming from the read power values affect the combined uncertainty less than the CF of the STD power sensor. But the uncertainties of the read powers are dependent on the STD and MON power sensors’ stabilities. The uncertainties coming from the reflection coefficient magnitude and phases are the components that affect the combined uncertainty the least, the same as in Table 3. Depending on the quality of the connectors, the effects of the reflection coefficient and phase uncertainty components on the combined uncertainty may increase.

Among the uncertainty components in Table 7, it is seen that the biggest contribution to the combined uncertainty comes from the CF of the STD power sensor, as is similar in Table 6. The power sensor stabilities and the connector’s quality can be more contributable to the combined uncertainty in the worst case.

**Figure 5:**
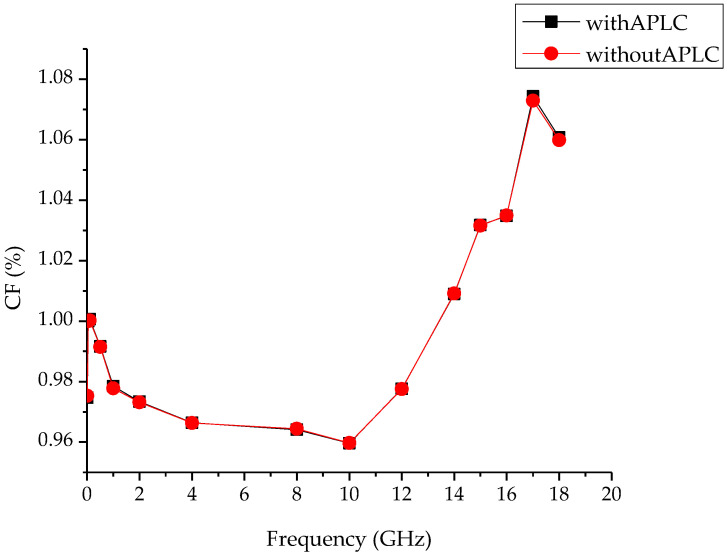
CF values of HP 8484A with and without APLC for −30 dBm.

The CF values with APLC and without APLC of the DUT are given in Figure 5 for the −30 dBm reference power level.

Figure 4 and Figure 5 show that the measurement results with APLC and without APLC at different power levels in coaxial power sensor calibrations produce a 0.1% difference from each other. This difference comes from the standard deviations in the measurements.

### 3.3. The WG Power Sensor Calibration by the DCTM at +8 dBm

In the WG power sensor calibration by the DCTM without APLC, the devices which were owned of TÜBİTAK UME were used. The frequency range was chosen between 110 GHz and 170 GHz for the WR6.5 connector size, and the PM5 (VDI) WG power sensor was used as a DUT power sensor with the proper power meter. In the measurement setup given in Figure 3, we used the E8257D (Agilent) model RF signal generator as the coaxial source to provide the necessary signal to the input of the frequency extender, the SGX37 (VDI) model frequency extender for access to the millimeter wave frequencies, a WG sensor designed by R&S and PTB as the reference (STD) power sensor with an appropriate voltmeter, a similar PM5 (VDI) model power sensor with a DUT power sensor as a monitor power sensor with an appropriate power meter, and a WR6.5 size Directional Coupler (DC) (VDI) as a power splitter. A power of 8 dBm with a ±2 dBm tolerance was preferred for calibration. The DCTM without the APLC method was used, because the frequency extender had no APLC input port at this calibration. Ten repetitive measurements with 5 (five) s intervals at each frequency were performed, and the waiting time for power level stabilizations between frequencies was 120 s. In order to determine the effect of the RF connector error on the measurement results, the operator rotated the DUT power sensor’s WG flange and the STD power sensor’s WG flange two times at 180° intervals, and performed two measurements. The measurement results of the WG RF power sensor calibration using the DCTM without APLC are given in Table 8.

The measurement uncertainties were calculated according to the GUM Law of Propagation method. The experimental uncertainty values of the STD power sensor were evaluated with PTB, and this uncertainty was used for the calculation of the uncertainty of the DUT WG power sensor [11].

Operator errors were minimized by using an automated measurement software developed in the C # platform for both coaxial and WG power sensors’ calibrations (mmWave Auto RF Power Measurement Software, R1.0, TUBITAK UME) [24]. The 5 GHz frequency step was chosen for the measurements because of the wide frequency range in this study. In order to see clearly the frequency response of the WG power sensor, small frequency steps can be suggested to researchers for future studies.

**Figure 6:**
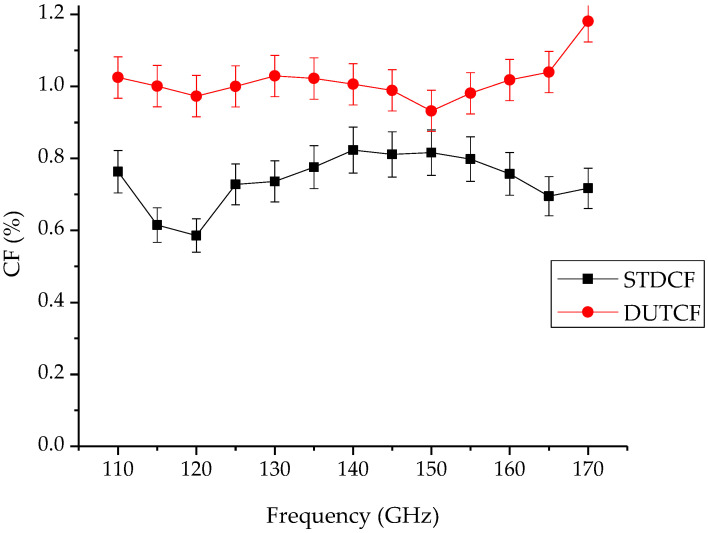
CF values of VDI PM5 (DUT) and PTB (STD) and uncertainty values of WG power sensor’s calibration.

The calculated CF results of the DUT WG RF power sensor are given in Figure 6 with the STD WG RF power sensor. The CF values of the DUT WG RF power sensor were calculated similarly to Equation (2). The STD WG RF power sensor uncertainties and the calculated uncertainties of the DUT WG RF power sensor are shown in Figure 6. It is shown that the calculated CF of the DUT WG RF power sensor and its uncertainties follow the STD WG RF power sensor’s values.

## 4. Conclusions

Although the earlier studies recommended the APLC for the DCTM, which is used in the reliable and accurate calibration of power sensors in many experiments, this study has shown that the calibration of coaxial power sensors could be performed without APLC at different power levels. For 0 dBm and −30 dBm power levels, the DCTM with APLC and the DCTM without APLC experimental coaxial power sensor calibration processes were performed in this study. The measurement results showed that the calculated CFs of the DUT power sensor with APLC and without APLC follow each other with %0.1 differences. These differences have come from the standard deviation of the measurements. These differences can be accepted when the differences are compared with extended uncertainties.

Based on these coaxial power sensor calibration results, it was decided that the DCTM without APLC can be used for the WG power sensor’s calibration. The WG RF power sensor’s calibration by the DCTM without APLC was carried out in this study. The calculated CF values of the DUT WG RF power sensor and the CF values of the STD WG RF power sensor were compared, and it has been shown that the calculated CF of the DUT WG RF power sensor and its uncertainties follow the STD WG RF power sensor’s values.

In this study, it has been shown that the WG power sensor’s calibration can be performed by using the DCTM without APLC either. The DCTM without APLC is suggested for future WG power sensor calibration studies.

## Figures and Tables

**Table 1:** Advantages and disadvantages of SM and DCTM.

Method	Advantages	Disadvantages
**SM**	Less devices required	Higher uncertainty, is achieved
	Less complicated measurement setup and an easier process to apply	RF signal source output is assumed to be the same as the DUT and STD power sensor connection
	Easier evaluation for CF and uncertainty	Vectorial impedance mismatch is not taken into account for CF evaluation
**DCTM**	Lower uncertainty is achieved than by SM	More devices used for measurement
	Vectorial impedance mismatch is taken into account for CF evaluation	More complicated measurement process and experienced operator required

**Table 2:** Measurement results of aoaxial RF power sensor calibration by DCTM with APLC and by DCTM without APLC for 0 dBm.

Freq. (GHz)	App. Pow. with APLC (mW)	CF_DUT_ (%) with APLC	Unc. of CF_DUT_ with APLC	App. Pow. without APLC (mW)	CF_DUT_ (%) without APLC	Unc. of CF_DUT_ without APLC
0.03	6.05	1.0089	0.0093	6.00	1.0062	0.0091
0.05	6.07	1.0000	0.0069	6.00	1.0000	0.0071
0.1	6.12	0.9902	0.0073	6.00	0.9918	0.0076
0.3	6.19	0.9821	0.0070	6.00	0.9833	0.0073
0.5	6.18	0.9758	0.0071	6.00	0.9761	0.0073
0.7	6.19	0.9679	0.0071	6.00	0.9681	0.0074
0.8	6.23	0.9621	0.0070	6.00	0.9620	0.0074
1	6.27	0.9438	0.0084	6.00	0.9444	0.0087
2	6.31	0.8913	0.0081	6.00	0.8913	0.0088
3	6.37	0.8636	0.0076	6.00	0.8625	0.0092
4	6.71	0.8923	0.0077	6.00	0.8890	0.0102
5	6.57	0.8626	0.0076	6.00	0.8599	0.0099
6	6.85	0.8478	0.0074	6.00	0.8430	0.0109
7	6.88	0.8195	0.0073	6.00	0.8153	0.0108
8	7.06	0.8246	0.0071	6.00	0.8172	0.0113

**Table 3:** The uncertainty component distribution for DUT CF uncertainty with APLC at 50 MHz, 0 dBm.

Uncertainty Component	% Contribution to Combined Uncertainty	Value
u_CF	0.999998558	1.18 × 10^−5^
u_P_D_	0.000432525	5.12 × 10^−9^
u_P_S_	0.000637804	7.56 × 10^−9^
u_P_mD_	0.000443155	5.25 × 10^−9^
u_P_mS_	0.000637684	7.55 × 10^−9^
u_Γ_D_	5.07342 × 10^−6^	6.01 × 10^−11^
u_Γ_S_	3.40824 × 10^−7^	4.04 × 10^−12^
u_Γ_PS_	2.97573 × 10^−5^	3.53 × 10^−10^
u_Θ_D_	8.70979 × 10^−18^	1.03 × 10^−22^
u_Θ_S_	5.42313 × 10^−9^	6.42 × 10^−14^
u_Θ_PS_	0.001298247	1.54 × 10^−8^

**Table 4:** The uncertainty component distribution for DUT CF uncertainty without APLC at 50 MHz, 0 dBm.

Uncertainty Component	% Contribution to Combined Uncertainty	Value
u_CF	0.999998618	1.24 × 10^−5^
u_P_D_	0.000432525	5.38 × 10^−9^
u_P_S_	0.000603905	7.52 × 10^−9^
u_P_mD_	0.00044295	5.51 × 10^−9^
u_P_mS_	0.000574432	7.15 × 10^−9^
u_Γ_D_	5.07342 × 10^−6^	6.31 × 10^−11^
u_Γ_S_	3.40824 × 10^−7^	4.24 × 10^−12^
u_Γ_PS_	2.97573 × 10^−5^	3.70 × 10^−10^
u_Θ_D_	8.70979 × 10^−18^	1.08 × 10^−22^
u_Θ_S_	5.42313 × 10^−9^	6.75 × 10^−14^
u_Θ_PS_	0.001298247	1.62 × 10^−8^

**Table 5:** Measurement results of coaxial RF power sensor calibration by DCTM with APLC and by DCTM without APLC for −30 dBm.

Freq. (GHz)	App. Pow. with APLC (mW)	CF_DUT_ (%) with APLC	Unc. of CF_DUT_ with APLC	App. Pow. without APLC (mW)	CF_DUT_ (%) without APLC	Unc. of CF_DUT_ without APLC
0.01	6.20	0.9747	0.0085	6.00	0.9753	0.0088
0.05	6.08	1.0000	0.0087	6.00	1.0000	0.0087
0.1	6.08	1.0006	0.0086	6.00	1.0002	0.0087
0.5	6.13	0.9916	0.0078	6.00	0.9915	0.0079
1	6.23	0.9785	0.0095	6.00	0.9778	0.0096
2	6.24	0.9734	0.0088	6.00	0.9732	0.0088
4	6.62	0.9664	0.0091	6.00	0.9663	0.0092
8	6.96	0.9641	0.0085	6.00	0.9644	0.0085
10	7.11	0.9596	0.0090	6.00	0.9597	0.0091
12	7.19	0.9776	0.0090	6.00	0.9775	0.0091
14	7.37	1.0090	0.0092	6.00	1.0091	0.0093
15	7.71	1.0317	0.0098	6.00	1.0316	0.0099
16	7.65	1.0348	0.0100	6.00	1.0349	0.0101
17	7.89	1.0743	0.0122	6.00	1.0729	0.0124
18	8.11	1.0607	0.0128	6.00	1.0598	0.0127

**Table 6:** Uncertainty components distribution for CF uncertainty with APLC at 50 MHz, −30 dBm.

Uncertainty Component	% Contribution to Combined Uncertainty	Value
u_CF	0.999996964	9.22 × 10^−6^
u_P_D_	4.98270 × 10^−10^	4.60 × 10^−15^
u_P_S_	0.001068914	9.86 × 10^−9^
u_P_mD_	5.10515 × 10^−10^	4.71 × 10^−15^
u_P_mS_	0.001068713	9.86 × 10^−9^
u_S_21_	0.001002775	9.25 × 10^−9^
u_Γ_D_	0.000006621	6.11 × 10^−11^
u_Γ_S_	0.000000438	4.04 × 10^−12^
u_Γ_PS_	0.000038221	3.53 × 10^−10^
u_Θ_D_	9.20930 × 10^−18^	8.49 × 10^−23^
u_Θ_S_	1.80037 × 10^−13^	1.66 × 10^−18^
u_Θ_PS_	0.001667523	1.54 × 10^−8^

**Table 7:** Uncertainty component distribution for CF uncertainty without APLC at 50 MHz, −30 dBm.

Uncertainty Component	% Contribution to Combined Uncertainty	Value
u_CF	0.667729504	8.91 × 10^−6^
u_P_D_	0.000437649	5.84 × 10^−9^
u_P_S_	0.000635127	8.47 × 10^−9^
u_P_mD_	3.54812 × 10^−10^	4.73 × 10^−15^
u_P_mS_	0.000642955	8.58 × 10^−9^
u_S_21_	0.744403318	9.93 × 10^−6^
u_Γ_D_	3.67510 × 10^−12^	4.90 × 10^−17^
u_Γ_S_	0.000002803	3.74 × 10^−11^
u_Γ_PS_	4.84677 × 10^−12^	6.47 × 10^−17^
u_Θ_D_	1.24165 × 10^−25^	1.66 × 10^−30^
u_Θ_S_	2.14791 × 10^−17^	2.87 × 10^−22^
u_Θ_PS_	0.000000207	2.76 × 10^−12^

**Table 8:** Measurement results of WG RF power sensor calibration by the DCTM without APLC.

Freq. (GHz)	P_S_ (mW)	P_D_ (mW)	P_mS_ (mW)	P_mD_ (mW)	Γ_S_	Γ_D_	Γ_DC_	Θ_S_ (rad)	Θ_D_ (rad)	Θ_DC_ (rad)	CF_STD_ (%)	UCF_STD_ (k = 2)	CF_DUT_ (%)	UCF_DUT_ (k = 2)
110	3.28	4.41	0.59	0.59	0.023	0.019	0.019	−2.99	−2.85	−2.27	0.763	0.059	1.025	0.080
115	1.84	3.19	0.37	0.40	0.013	0.004	0.020	2.35	2.67	−2.92	0.615	0.048	1.001	0.078
120	3.07	5.55	0.57	0.63	0.019	0.013	0.019	2.28	2.52	2.44	0.586	0.046	0.973	0.076
125	3.81	5.01	0.56	0.54	0.010	0.012	0.020	1.98	−3.12	2.79	0.728	0.057	1.000	0.078
130	3.78	5.14	0.57	0.55	0.026	0.021	0.018	1.98	2.24	2.63	0.736	0.057	1.029	0.080
135	2.77	3.48	0.41	0.39	0.010	0.010	0.021	2.31	−2.95	2.50	0.776	0.060	1.022	0.079
140	3.18	3.65	0.45	0.43	0.018	0.023	0.034	−2.49	−2.12	2.62	0.823	0.064	1.006	0.078
145	3.21	4.00	0.46	0.47	0.107	0.094	0.195	2.43	2.41	2.26	0.811	0.063	0.989	0.077
150	2.73	3.09	0.40	0.39	0.011	0.019	0.030	−0.74	−0.50	1.96	0.816	0.063	0.932	0.072
155	4.51	5.63	0.71	0.73	0.017	0.019	0.195	0.09	0.13	2.26	0.798	0.062	0.981	0.076
160	3.03	4.04	0.57	0.57	0.020	0.020	0.026	0.94	1.14	1.96	0.757	0.059	1.018	0.079
165	0.95	1.45	0.21	0.22	0.021	0.020	0.034	2.11	2.55	2.37	0.695	0.054	1.040	0.081
170	1.57	2.61	0.43	0.43	0.015	0.015	0.028	−3.00	−2.46	2.34	0.717	0.056	1.181	0.092

## Data Availability

The data presented in this study are available on request from the corresponding author.
